# Bacterial Biofilm Control by Perturbation of Bacterial Signaling Processes

**DOI:** 10.3390/ijms18091970

**Published:** 2017-09-13

**Authors:** Tim Holm Jakobsen, Tim Tolker-Nielsen, Michael Givskov

**Affiliations:** 1 Costerton Biofilm Center, Department of Immunology and Microbiology, University of Copenhagen, 2200 Copenhagen, Denmark; ttn@sund.ku.dk (T.T.-N.); mgivskov@sund.ku.dk (M.G.); 2 Singapore Centre on Environmental Life Sciences Engineering, Nanyang Technological University, Singapore 637551, Singapore

**Keywords:** quorum sensing, cyclic-di-GMP, anti-virulence, small RNAs, *Pseudomonas aeruginosa*, biofilm life-cycle, cell signaling

## Abstract

The development of effective strategies to combat biofilm infections by means of either mechanical or chemical approaches could dramatically change today’s treatment procedures for the benefit of thousands of patients. Remarkably, considering the increased focus on biofilms in general, there has still not been invented and/or developed any simple, efficient and reliable methods with which to “chemically” eradicate biofilm infections. This underlines the resilience of infective agents present as biofilms and it further emphasizes the insufficiency of today’s approaches used to combat chronic infections. A potential method for biofilm dismantling is chemical interception of regulatory processes that are specifically involved in the biofilm mode of life. In particular, bacterial cell to cell signaling called “Quorum Sensing” together with intracellular signaling by bis-(3′-5′)-cyclic-dimeric guanosine monophosphate (cyclic-di-GMP) have gained a lot of attention over the last two decades. More recently, regulatory processes governed by two component regulatory systems and small non-coding RNAs have been increasingly investigated. Here, we review novel findings and potentials of using small molecules to target and modulate these regulatory processes in the bacterium *Pseudomonas aeruginosa* to decrease its pathogenic potential.

## 1. Introduction

Over the years, research has provided increased insight into the mode of life of bacteria, in turn presenting important challenges in the treatment of infectious diseases. Besides the increasing resistance to antibiotics, it has been shown that, where antibiotics have proven to be particularly effective in controlling acute infections, chronic infections are likely to withstand concentrations of antibiotics that would otherwise eradicate the infectious organism when grown as traditionally laboratory cultures. One major breakthrough in understanding the resilience of bacteria in chronic infections came with the discovery that bacteria generally live together in aggregated communities, called biofilms. Up until the 1970s, the general understanding of bacteria were that they live as single organisms (planktonic mode of life), whereas now the biofilm mode of growth is considered the favored life form of bacteria in their environments [1,2]. A highly elevated resistance patterns towards antibiotics and the immune system, compared with planktonic cells has been shown for infectious pathogenic bacteria living in said biofilms [3,4,5], which obviously has increased the attention towards this bacterial life form extensively. There are several reasons for the biofilm tolerance, including slow growth and the presence of an extracellular matrix consisting of various biopolymers. The formation and proliferation of a biofilm with its surrounding matrix are influenced by a multitude of factors including several complex regulatory systems that have shown to be involved in different stages of the biofilm life-cycle. For the Gram-negative opportunistic pathogen *Pseudomonas aeruginosa*, several have been identified including the cell–cell communication system Quorum Sensing (QS), the secondary messenger bis-(3′-5′)-cyclic-dimeric guanosine monophosphate (c-di-GMP or cyclic-di-GMP) and, more recently, the Gac/Rsm cascade.

*P. aeruginosa* can cause both acute and chronic infections, however, it is seldom found to cause severe infections in healthy individuals. However, it is found in relation to otitis media [6], periodontitis [7], and keratitis [6]. It is a major human pathogen in relation to immunocompromised patients, and it targets individuals suffering from, e.g., cancer undergoing chemotherapy, HIV patients, neoplasia and burn victims [8,9], and it is the most common airway pathogen in patients with the genetic hereditary disease, cystic fibrosis (CF) [10,11]. Specifically, the ability to cause chronic infections makes *P. aeruginosa* a problematic pathogen. This particularly applies to patients with indwelling catheters, inserted foreign bodies, people with leg and foot ulcers, and CF, where it is found in more than 80% of adults carrying the disease. In relation to CF, early aggressive antibiotic treatment is important to avoid an intermittent *P. aeruginosa* infection evolving into a chronic condition, making it impossible to eradicate. When a chronic infection has been established, the treatment is changed to suppressive maintenance therapy. Colonization, which involves formation of biofilms, increases the protection against administered antibiotics and the antimicrobial properties of the host immune systems.

**Figure 1:**
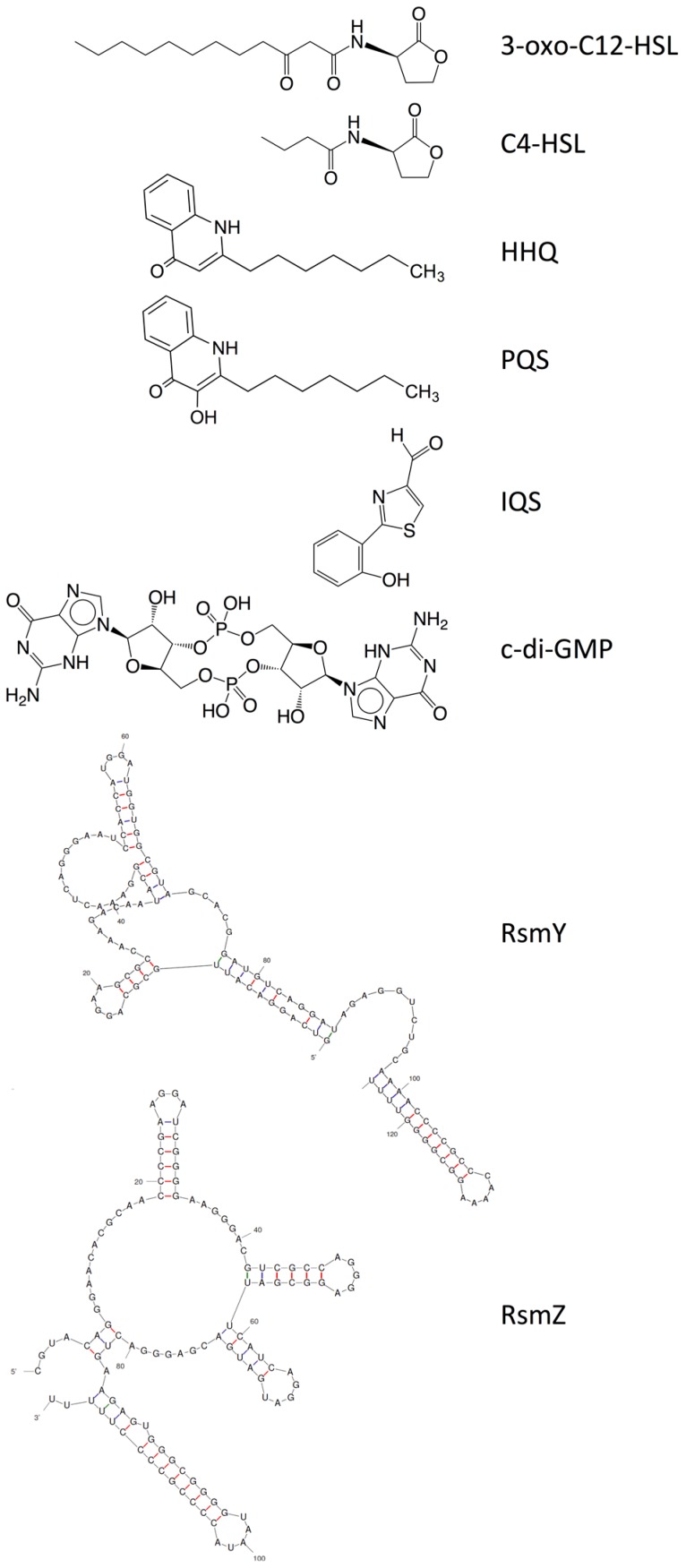
Structures of the native signal molecules of QS; 3-oxo-C12-HSL, C4-HSL, PQS, HHQ and IQS of *P. aeruginosa* as well as the internal signal molecule c-di-GMP of Gram-negative bacteria and predicted secondary structures generated using MFOLD (multiple fold) (http://mfold.rna.albany.edu/?q=mfold/RNA-Folding-Form) [15] of the non-coding regulatory small RNAs RsmY and RsmZ involved in the Gac/Rsm cascade from *P. aeruginosa* and *P. flourescence*. 3-oxo-C12-HSL: *N*-(3-oxododecanyol)-l-homoserine lactone, C4-HSL: *N*-butanoylhomoserine lactone, HHQ: 2-heptyl-4-hydroxyquinoline, PQS: 2-heptyl-3-hydroxy-4-guinolone, IQS: 2-(-hydroxyphenyl)-thiazole-4-carbaldehyde, c-di-GMP: bis-(3′-5′)-cyclic-dimeric guanosine monophosphate.

Increased knowledge of essential regulatory systems involved in the biofilm life-cycle has culminated in the awareness of the possibility of attenuating bacteria’s biofilm developing capacity. This reduces bacterial resilience and makes them more susceptible to subsequent combinatorial antibiotic treatments including the antimicrobial activities of the innate immune system. Hentzer et al. [12,13] delivered the first proof of concept regarding QS inhibition as an antimicrobial principle with chemically modified brominated furanones. For the first time, treatment of an infection with a compound that does not interfere with growth of the infecting bacteria was shown to display antimicrobial activity in vivo. This discovery has led to an ongoing search for QS inhibitors (QSIs) with the potential for medicinal application as well as a search for other targets that can potential be used in anti-biofilm treatment strategies. Targeting the biofilm and virulence without killing the bacteria has gained considerable attention as an anti-pathogenic strategy [14] and the present review turns to such investigations regarding small compounds capable of inhibiting cellular regulatory systems like, the QS systems (i.e., QSIs), c-di-GMP as well as the Gac/Rsm cascade in *P. aeruginosa* from both natural and synthetic sources. Figure 1 shows the native signal molecules of the QS and the c-di-GMP systems together with the secondary structures of the small regulatory non-coding RNAs RsmY and RsmZ, which are involved in the Gac/Rsm cascade. The purpose of this review is: (I) to present a overview of the three regulatory systems, QS, c-di-GMP and the Gac/Rsm cascade with focus on potential connections to the biofilm life-cycle; (II) to present an representative overview of natural and synthetic small molecule inhibitors of one of the three mentioned systems; and (III) to discuss future perspectives of small molecule inhibitors as potential treatments of biofilm-related infections.

## 2. Bacterial Biofilms

It has been generally accepted that the formation of biofilms in the host organism correlates with the development of chronic infections [16]. Formation of in vitro biofilms can be described as proceeding through different stages [17], to which *P. aeruginosa* has been a model organism. The development of biofilms has been suggested to proceed according to an underlying genetic program [18,19]. However, in vitro observations of gene expression profiles support the view that biofilm formation primarily involves adaptation to nutritional and environmental conditions [20,21,22]. The primary characterization of the processes and factors involved in formation of biofilm is based on in vitro investigations because studies of biofilms in living organisms have their obvious technical challenges.

### The Protective Biofilm Matrix of P. aeruginosa

The biofilm construction is made of microcolonies surrounded by a matrix consisting of extracellular polymeric substances (EPS). The EPS are primarily self-generated by the bacteria and consist of polysaccharides, proteins, lipids, extracellular DNA (eDNA) and biosurfactants [23]. In addition, to the EPS generated by the bacteria parts of the EPS can be adopted from the host organism which has been shown to be the case for eDNA which provides structural stability to the biofilm [24,25,26,27]. Treatment of an in vitro biofilm with DNase has been shown to inhibit the biofilm formation as well as to disperse young biofilms [25]. Contrary to this, DNase treatment of “older” and mucoid in vitro biofilms has not had any substantial effect. However, treatment with DNase was approved for human use in the late 60 s [28] and subsequent studies of CF patients treated with nebulized DNase showed a significant improvement of lung function [3,29,30,31]. Today, the DNase Pulmozyme^®^ (Genentech, South San Francisco, CA, USA) is used in the treatment of chronic biofilm infections in the lungs of CF patients [3]. Exopolysaccharides also provide structural stability, as well as contribute to the formation of biofilms, of which *P. aeruginosa* produce three different kinds: alginate, Psl and Pel. Alginate is not generally produced under normal environmental conditions, whereas it is induced by a *mucA* mutation (mucoid phenotype), which, in turn, is documented to take place primarily in chronic infections, e.g., the lungs of CF patients [32]. The formation of the exopolysaccharides, Pel and Psl, is influenced by the Gac/Rsm cascade. Both contribute to the biofilm architecture [33,34,35], while *pel* encodes an uncharacterized exopolysaccharide [36], the gene products of *psl* direct the synthesis of a characterized pentasaccharide [37]. In addition, they have recently been suggested to have redundant functions in a mature biofilm [38].

Individual cells can detach from the biofilm by dispersion. This function is very dependent on changes in the environmental conditions such as starvation [39], change in carbon source [40], increasing nitric oxide levels [41] and/or iron limitations [42]. These environmental stimuli and signals are detected by sensors in the individual cells in the biofilm leading to events regulating the intracellular level of c-di-GMP with the important role of making a switch between biofilm formation and dispersal [43]. In the next chapter factors involved in the protection as well as structural stability of the biofilm which are controlled by the QS, c-di-GMP system or the Gac/Rsm cascade will be described.

## 3. Cellular Regulatory Systems Involved in the Biofilm Life-Cycle

**Figure 2:**
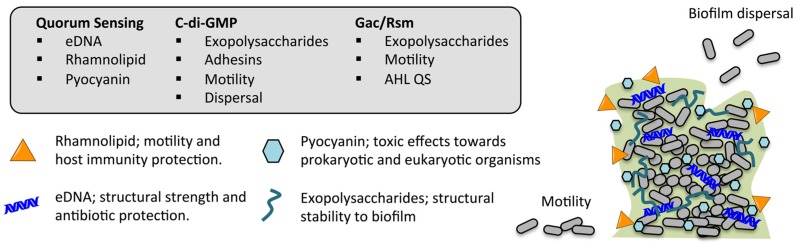
Inter-cellular regulatory systems involved in *P. aerugionsa* biofilm life-cycle; quorum sensing, c-di-GMP and Gac/Rsm cascade with examples of essential factors being produced under the control of these particular systems. AHL: *N*-acyl-l-homoserine lactone; QS: quorum sensing.

Several cellular regulatory systems are involved in the complex regulation of the biofilm life-cycle in *P. aeruginosa*, with c-di-GMP, Gac/Rsm cascade and QS being some of the most important and relevant key actors both for the formation, structural stability and protective role of the biofilm (Figure 2). Production of external factors involved in the biofilm, such as eDNA, pyocyanin, rhamnolipid and exopolysaccharides are under the influence of either one or more of these regulatory systems also making some connections. Each of the systems will be described in the following with a focus on the connection to the biofilm life-cycle.

### 3.1. Quorum Sensing and Its Role in the P. aeruginosa Biofilm

*P. aeruginosa* is by far the most studied organism with respect to the *N*-acyl-l-homoserine lactone (AHL) mediated QS. Depending on the growth conditions, it is estimated that 4–6% of the total number of genes in *P. aeruginosa* is controlled by QS [12,44,45]. Among these are genes that encode an arsenal of virulence factors, such as proteases [46], elastases [47], exoenzymes [48,49], the siderophores pyochelin and pyoverdin [50], the glycolipid rhamnolipid [51], and genes involved in iron metabolism [22]. The influence of QS on biofilm formation has been debated over the years. Several studies have shown biofilm formation of QS-deficient *P. aeruginosa* strains in different in vitro biofilm settings when compared to a WT biofilm the QS-deficient biofilm tend to more homogenous and fragile [52]. Especially, the three partially or complete QS regulated factors, eDNA, rhamnolipid and pyocyanin, have been shown to influence the biofilm architecture and provide an inherent protection from external factors, such as host immunity and antibiotic therapy.

The QS controlled production of eDNA [24,27,53] provides both structural strengths to the biofilm and increase the antimicrobial tolerance. In addition, it has been shown to be a contributing factor in blockage of the respiratory airways in CF patients by generating high viscosity of the sputum [54]. Same study found that sputum from CF patients infected with *P. aeruginosa* contained 3–14 mg/mL contrary to zero eDNA in non-CF patients [54]. DNA is a highly anionic polymer and its role in increasing the antibiotic tolerance is believed to be by binding to positively charged antibiotics such as aminoglycosides and antimicrobial peptides [55,56,57]. In addition, high amounts of DNA has been shown to have an cation-chelating effect causing cell lysis resulting in release of cytoplasmic contents, including DNA [58]. It has been shown that during biofilm infections, the eDNA incorporated into a biofilm may also be provided by lysed cells from the host defense system [59]. A recent study suggests both internally produced and exogenously added eDNA as the main reason for the increased antimicrobial protection [60].

Phenazines are a group of pigmented molecules produced by a number of bacteria, including *Pseudomonas* spp. Many of the phenazines display toxic effects towards prokaryotic and/or eukaryotic organisms, which may confer an advantage for the producers in the competition for survival. The most studied phenazine is pyocyanin, which is only found to be produced by *P. aeruginosa*. Production of pyocyanin is regulated by the PQS system with expression of the *phzA-G* operon resulting in pheniine-1-carboxylic acid (PCA), which is then modified to pyocyanin regulated by the *phzM* gene. Pyocyanin is regarded as one of the major virulence factors of *P. aeruginosa* contributing to both acute and chronic infections [61,62]. In vitro studies have shown that pyozyanin targets a range of different cellular functions. To name some, pyozyanin damages epithelial cells [62], suppresses lymphocyte proliferation [63], and inactivates the protease inhibitor alpha1, resulting in tissue damage from the increased level of endogenous proteases [64]. Furthermore, it alters calcium signaling in airway epithelial cells [65], inhibits prostacyclin production by pulmonary endothelial cells [66], and limits growth of T lymphocytes by targeting the release of interleukin-2 [67]. Pyocyanin have a potential role in the *P. aeruginosa* biofilm formation by both promoting eDNA release and by binding to eDNA. The release of eDNA has been shown to be induced by cell lysis due to hydrogen peroxide (H_2_O_2_). The presence of pyocyanin increases the formation of H_2_O_2_ which thereby leads to an increase cell lysis [68]. The direct binding of pyocyanin to the nitrogenous base pairs of DNA influence the cell surface hydrophobicity and thereby the aggregation of cells increasing the structural stability of the biofilm [69].

**Figure 3:**
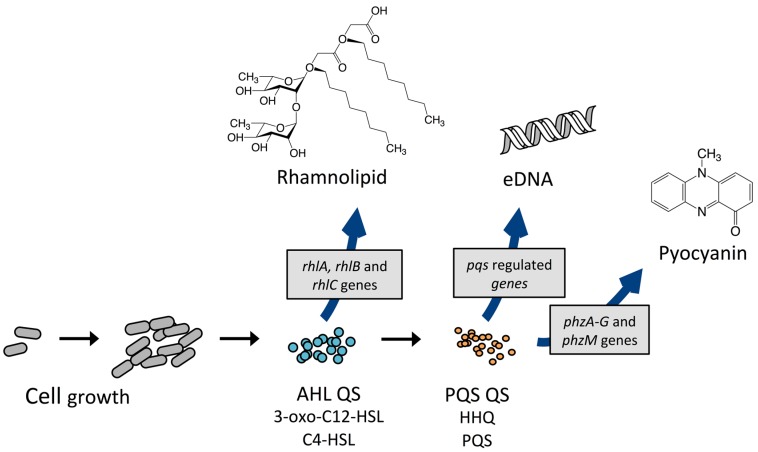
Production of QS regulated rhamnolipid, pyocyanin and eDNA by *P. aeruginosa*. QS: quorum sensing; AHL: *N*-acyl-l-homoserine lactone; 3-oxo-C12-HSL: *N*-(3-oxododecanyol)-l-homoserine lactone; C4-HSL: *N*-butanoylhomoserine lactone; HHQ: 2-heptyl-4-hydroxyquinoline; PQS (pseudomonas quinolone signal): 2-heptyl-3-hydroxy-4-guinolone.

Another QS regulated factor considered to be a key virulence determinant is rhamnolipids, a rhamnose-containing glycolipid biosurfactant (see Figure 3) [70]. *P. aeruginosa* produces two main classes of rhamnolipids; mono- and di-rhamnolipids [71], which are encoded by three genes, carried on two distinct operons: one operon carries the *rhlAB* genes (*rhlA*, PA3479) (*rhlB*, PA3478) and the other *rhlC* (PA1131). The *rhlAB* operon encodes a rhamnosyltransferase which takes part in the formation of mono-rhamnolipid and *rhlC* encode a second rhamnosyltransferase that converts mono-rhamnolipid to di-rhamnolipid [72,73]. Rhamnolipid is a heat stable hemolysin [74] with multiple properties. It has been shown to rapidly lyse neutrophils, macrophages, and different animal cells [75,76,77]; to be involved in swarming motility [78]; to shape the structure of biofilms [79]; and to possess antimicrobial activity against different bacteria, such as *S. aureus*, *Klebsiella pneumonia*, *Serratia marcescens* and *Bacillus subtilis* with MICs ranging from 0.5 to 32 μg/mL, as well as the fungi *Penicillium funiculosum* and *Fusarium solani* with MICs of 16 and 75 μg/mL, respectively [80]. The antimicrobial properties of rhamnolipids may give *P. aeruginosa* an advantage in relation to niche colonization in the environment, as well as in the competition with other bacteria in, e.g., the lungs of CF patients [81]. In addition, a study using biopsies of nasal mucosa has shown that rhamnolipid contribute to the establishment and maintenance of a *P. aeruginosa* infection in the lungs of CF patients by entering the epithelial cells and disrupt tight-junctions, which, in turn, leads to paracellular invasion of *P. aeruginosa*. This prevents further clearing of mucus in the lungs by causing ciliostasis [82,83]. By killing polymorphonuclear leukocytes (PMNs), rhamnolipid protects against the immune defense thereby increasing the potential for survival [75]. This function has been termed “the rhamnolipid shield” [84]. It has been shown that the production of rhamnolipid in in vitro biofilms is relatively low [85,86], however, Alhede et al. demonstrated an increase in rhamnolipid production in a biofilm by exposure to PMNs and suggests that a *P. aeruginosa* biofilm can sense the presence of PMNs and reacts by mounting a rhamnolipid shield [85]. How the bacteria sense the PMNs is still an unanswered question that needs to be unraveled. The rhamnolipid protection has further been demonstrated in different mouse models with an *rhlA* mutant showing significantly faster clearing compared to a WT *P. aeruginosa* [87]. It has further been shown that lysis of PMNs results in an enhancement of biofilm formation from release of PMN DNA which then functions as eDNA [59,85]. This means that when the biofilm infection is not eradicated by the immune defense, the subsequent incorporation of eDNA liberated from PMNs in the biofilm matrix facilitates a further increase in structural stability of the colony as well as increase in resistance towards positively charged antibiotics [60]. The production of rhamnolipid and pyocyanin has also been shown to be under the control of the IQS system, which was recently identified as a new class of QS signal molecule in *P. aeruginosa.* The system was shown to be tightly controlled by the las system under normal growth conditions and during phosphate limiting conditions to take over for a non-functioning las system by activating the PQS system [88]. As mutations in *lasI* or *lasR* genes have frequently been found in *P. aeruginosa* clinical isolates [89,90,91,92,93], the IQS system can function as an alternative signal mechanism to maintain virulence gene expression in *P. aeruginosa* during chronic infections.

### 3.2. Bis-(3′-5′)-cyclic-dimeric Guanosine Monophosphate (Cyclic-di-GMP) and Its Role in P. aeruginosa Biofilm

The nucleotide-based molecule c-di-GMP has attracted a lot of attention over the last decade because of its major role as an intracellular secondary signal molecule in the switch between the biofilm mode of growth and motility. Studies of this relatively new signaling pathway has demonstrated an extremely complex regulatory system that seems to be involved in many important factors favoring survival potentials by changing the lifestyle of pathogens such as *P. aeruginosa*. The lifestyle switch is dependent on the intracellular concentration of c-di-GMP, with low levels favoring the expression of factors involved in motility, and high levels favoring the sessile lifestyle by increasing the expression of adhesion factors and extracellular matrix components [94,95,96].

The level of c-di-GMP in the bacteria is regulated by actions of two types of enzymes: diguanylate cyclase (DGC) enzymes, which contain GGDEF domains and synthesize c-di-GMP from two molecules of GTP, and phosphodiesterase (PDE) enzymes, containing an EAL or HD-GYP domain, which degrades c-di-GMP. PDEs with an EAL domain degrade c-di-GMP to the linear dinucleotide pGpG [97] and the HD-GYP domain catalyzes degradation of c-di-GMP to two GMP molecules [98]. In addition, several proteins capable of both synthesis and degradation of c-di-GMP, by containing both GGDEF and EAL domains, have been identified [96]. By genome sequence analysis of *P. aeruginosa* PAO1 and PA14, 39 genes encoding proteins with either one or both DGC and PDE domains have been identified [99]. Even though the system has been widely studied in recent years, many details are still unknown, particularly the identification of c-di-GMP binding proteins. A recent study used a chemical proteomics approach in the attempt to identify c-di-GMP binding proteins. It determined over 100 such proteins, including several that had already been previously identified, such as WspR, a DGC prone to allosteric feedback inhibition; BifA, a protein with a degenerated GGDEF domain; FimX, which harbors a catalytically inert GGDEF-EAL hybrid domain; and an additional 24 proteins involved in flagellum- or pili-mediated chemotaxis [100].

The importance of c-di-GMP in biofilm formation is exemplified by the identified factors regulated by c-di-GMP such as Psl, Pel and alginate exopolysaccharides [101,102,103]; Cup fimbrael adhesins [104]; surface adhesin CdrA [105]; and type IV pili [104], as well as in a number of studies showing changes in biofilm formation concurrent with changes in the intracellular c-di-GMP level [105,106].

C-di-GMP is a positive regulator of the production of several adhesins in *P. aeruginosa*. Type IV pili synthesis was shown to be regulated by c-di-GMP in a manner that depend on the proteins PilZ and FimX which have c-di-GMP binding domains [107,108]. Moreover, evidence was provided that synthesis of Cup fimbriae is regulated by c-di-GMP [109,110,111]. Expression of *cupA* was found to depend on the proteins WspR, MorA, and PA1120 which have either GGDEF or c-di-GMP binding domains [104,112]. The PDE RocR was shown to be involved in synthesis of CupB and CupC fimbriae [109,111], and the PDE PvrR was found to be involved in the synthesis of CupD fimbriae [104,110]. Furthermore, CupA has been shown to be involved in SDS-induced auto-aggregation dependent upon increased intracellular levels of c-di-GMP mediated by the SiaD DGC [113,114]. In addition, expression of the large surface protein CdrA, that has been shown to tether *P. aeruginosa* cells to the Psl polysaccharide, is positively controlled at the transcriptional level by c-di-GMP [105].

Transcription of the *psl* polysaccharide genes was also found to be positively regulated by c-di-GMP [115,116]. In addition, expression of *psl* was found to be regulated at the post-transcriptional level through binding of RsmA protein to the *psl* mRNA when RsmA is not sequestered by the small RNAs RsmY and RsmZ [115]. Furthermore, Irie et al. [117] reported that the Psl polysaccharide stimulates c-di-GMP synthesis by the SiaD and SadC DGCs. Elevated concentrations of c-di-GMP then lead to increased production of Psl and other components of the biofilm matrix. The extracellular polysaccharide thus promotes biofilm formation via a unique positive feedback regulatory circuit, which may play a role for recruitment of planktonic bacteria to adhere to an existing biofilm.

Synthesis of Pel polysaccharide was found to be regulated by c-di-GMP both at the transcriptional and post-translational level. Microarray analysis performed on a *P. aeruginosa* wild-type strain and its isogenic *wspF* mutant (which has elevated c-di-GMP levels) suggested increased transcription of the *pel* genes in the *wspF* mutant [116]. Accordingly, Hickman & Harwood [102] found that c-di-GMP binds to the transcriptional regulator FleQ and thereby induces transcription of the *pel* genes. In addition, Lee et al. [103] presented evidence that Pel polysaccharide synthesis is activated at the post-translational level by binding of c-di-GMP to the protein PelD which is part of the membrane associated Pel synthase.

Alginate synthesis is also positively regulated by c-di-GMP. The membrane-anchored protein, Alg44, which is part of the alginate synthase, contains a c-di-GMP-binding PilZ domain [118,119,120,121]. Stimulation of Alg44 activity via the PilZ domain evidently occurs through binding of c-di-GMP synthesized by the membrane anchored DGC MucR [101].

Several aspects indicate that c-di-GMP is involved in changing an infection towards a chronic state. As mentioned previously, high c-di-GMP levels have been shown to switch secretion systems from T3 to T6. In addition, the mucoid phenotype, which is associated with chronic infections, is to some extent regulated by c-di-GMP. Moreover, *P. aeruginosa* CF isolates that displayed a rugose small-colony variant (RSCV) phenotype were found to have increased levels of c-di-GMP and *pel* and *psl* expression, and it was suggested that these RSCVs may contribute to increased persistence of the airway infection [122]. Many of the RSCVs isolated from CF patients were demonstrated to be *wspF* mutants which overexpress the DGC WspR. In addition, Malone et al. [123] have demonstrated that some *P. aeruginosa* RSCVs isolated from CF patients have a mutation in the *yfi* gene so that they produce a high level of c-di-GMP due to high activity of the YfiN DGC.

### 3.3. Two-Component Systems and Small RNAs

Upstream of the AHL and PQS systems, which can be termed the central parts of the QS system, is a two-component transduction system, the Gac system, initially identified in *P. syringae* [124]. Over 60 two-component systems have been identified in *P. aeruginosa*, and Gac is the most intensively studied. It consist of a transmembrane sensor kinase, GacS, that autophosphorylates a conserved histidine residue and this phosphate is transferred to its cognate regulator, GacA, that affects the transcription of the two small RNAs (sRNAs), RsmZ and RsmY [125]. These two sRNAs bind to the CsrA homolog RsmA, a global post-transcriptional regulatory protein that suppresses different target genes which are involved in QS, extracellular products, biofilm formation, and motility [126]. RsmA has a negative effect on the production of C4-HSL and 3-oxo-C12-HSL [127,128] and RsmY and RsmZ opposes this negative effect of RsmA, leading to increased production of virulence factors.

A second CsrA homolog has recently been identified in *P. aeruginosa* [129,130]. The RsmF (also called RsmN) mRNA binding protein has several binding targets in common to RsmA, including RsmY and RsmZ, however the binding affinities for RsmF are significantly lower for these sRNAs compared to RsmA. On the other side RsmF show different binding properties to RsmA by e.g., not being able to bind to *pslA* such as RsmA [129].

In addition to GacS, two other sensor kinases have been identified to modulate gene expression via GacA. RetS (Regulator of Exopolysaccharide and Type III Secretion) and LadS (Lost Adherence Sensor) [131] were classified almost 10 years ago. In the search for the effect of RetS on GacS/A, Goodman et al. [132] demonstrated that the effect is achieved by the formation of heterodimers between RetS and GacS, in turn leading to a blockage of the autophosphorylation of GacS, indicating that RetS is an antagonist of GacS. LadS has the opposite function by activating the expression of RsmY and RsmZ through GacA [131,133,134]. A recent study report a novel RsmA binding RsmY/RsmZ-type sRNA, termed RsmW shown not to be regulated by GacA contrary to *rsmY* and *rsmZ* expression and up-regulated under biofilm growth and in nutrient limited conditions [135].

In addition to the role of the Gac system in regulating QS, it is recognized from in vitro observations as being involved in a switch between planktonic and biofilm mode of growth by regulating the exopolysaccharides Pel and Psl [115,136] and type IV pili. The Gac system is also recognized to functioning as a switch between acute and chronic infections by regulating expression of genes being associated with type-III and type-VI secretion system (T3SS and T6SS) via RsmA [115]. From in vitro studies, acute infections are normally associated with a high production of virulence factors and secretion via the T3SS, whereas chronic infections, very likely involving the biofilm mode, appear less virulent with expression of the T6SS [137,138]. From studies with mutants, the switch has been suggested to be facilitated by the two sensors, RetS and LadS. Mutants of *retS* and *rsmA* show similar phenotypes with overproduction of exopolysaccharides, enhanced biofilm, reduced expression of T3SS, and diminished type IV pili motility [132,139]. Contrary to this, a *ladS* mutant reduced biofilm formation and enhanced expression of T3SS [140]. Despite extensive investigations into the Gac system, the signal—or signals—detected by the sensor kinases, GacS, LadS and RetS, triggering the phosphorylation response has not been identified. Finding the activators of sensor kinases is generally an unsolved issue for the vast majority of two-component systems [141]. However, the identification of such signals would be of great interest because it enables a way to control the behavior and switching bacterial lifestyle, which could be useful in relation to treatment of infections.

### 3.4. Interconnections between the Regulatory Systems

Several factors have shown to be regulated by QS, c-di-GMP and the Gac/Rsm cascade, in turn indicating some common traits in these three systems. A recent study has added another common trait by indicating that the Gac/Rsm cascade regulates genes encoding iron uptake, and thereby siderophore production, through modulation of the intracellular level of c-di-GMP [142]. High c-di-GMP levels as well as activation of the Gac system induce biofilm formation and development of chronic infections (signaled by expression of the T6SS) while reducing motility and acute virulence (signaled by expression of the T3SS). As mentioned earlier, an activated Gac system is generally associated with reduced virulence. However, the increased production of QS controlled virulence factors which will take place as a result of an activated Gac/Rsm cascade questions this association. Any interaction between the central parts of the QS system and c-di-GMP systems has not been convincingly demonstrated, whereas a direct interaction between the Gac/Rsm cascade and the c-di-GMP system has been documented in a few studies. The first hint came from the Filloux group showing a link between c-di-GMP and RetS pathways which was presented by studying the switch between T3SS and T6SS [143]. They showed a *retS* mutant to display high levels of c-di-GMP, that the production of T3SS and T6SS can be switched by modulating the c-di-GMP levels, and that functional RsmY and RsmZ are required for the c-di-GMP response. How c-di-GMP influence the Gac/Rsm cascade is unclear from this study, however, the c-di-GMP dependent switch requires a functional Gac system in order to have any effect. Later the group showed a link between the systems by showing that the DGC SadC is repressed by RsmA [144].

## 4. Signal Perturbation by Small Molecules

The importance of QS, c-di-GMP and the Gac/Rsm cascade in the biofilm life-cycle has lead to substantial attention to the potential of modulate these systems by small molecules. A large number of different methods have been developed to identify and investigate the potential of cell signaling inhibitors. Screening methods used extensively at The Costerton Biofilm Center to investigate small molecules for signaling modulating activity is live monitor bacteria with a promoter of interest fused to genes encoding green fluorescent protein (GFP). Our reporter strain repertoire consists, among others, of strains able to detect modulations of the *las*- [13] and *rhl*-encoded [145] QS systems, the Gac/Rsm cascade with *rsmY* and *rsmZ* reporter strains [146] and our recently constructed c-di-GMP reporter [147]. The transcriptionally c-di-GMP reporter is constructed by fusing the DGC *cdrA* promoter to *gfp*. To increase the c-di-GMP content for better detection potential the strain is a *wspF* mutant and in addition, the monitor strain harbors *pelA* and *pslBCD* mutations to avoid clumping during growth. The use of live reporter systems is a great tool for an initial screening of potential inhibitors, and has led to the identification many active extracts and compounds. High throughput docking analysis is a widely used method for both detecting potential QS and c-di-GMP inhibitors. This method is often used to detect analogs of the natural signaling molecules, however, a major challenge using this approach is the risk that identified compounds do not freely pass bacterial membranes, which is particular evident with investigation of c-di-GMP analogs and the inability of the natural signal molecule to freely pass membranes.

By using QS-deficient *P. aeruginosa* strains the most important implications in relation to *P. aeruginosa* infections, is that blocking of the QS system restores proper action of the innate immune system with subsequent clearance of the infecting bacteria [52]. Several in vivo studies have shown reduced virulence in *P. aeruginosa* strains with single or double mutants of *lasI*, *rhlI*, *lasR or rhlR* compared to wild-type *P. aeruginosa* by using different models such as burned-mouse model [148], an adult mouse acute-pneumonia model [149], neonatal mouse model of pulmonary infection [150], rat model of chronic lung infection [151], and a foreign-body infection mouse model [152]. In addition, it has more recently been shown that a *pqsR* mutant display a reduced mortality rate in mice [153].

The first in vitro study of *P. aeruginosa* biofilm dispersal by exposure to a small external compound was based on nitric oxide (NO). The NO donor sodium nitroprusside (SNP) was shown to increase dispersal of a *P. aeruginosa* biofilm together with an increase in removal of an established biofilm by combination treatment of SNP and different conventional antimicrobial agents [41]. The first proof of concept of induced dispersal as a potential treatment strategy was delivered by us using a foreign-body infection model in mice. By using a construct where a reduction in c-di-GMP level in a *P. aeruginosa* strain is under the control of the PDE Yhjh from *E. coli* dispersal was generated in both established *P. aeruginosa* in vitro and in vivo biofilms [106].

### 4.1. Compounds Modulating Quorum Sensing (QS) Signaling

The last two decades of searching for chemistry capable of interfering with QS has revealed that such molecules can be found among natural produced secondary metabolites. The structure of the natural halogenated furanones produced as a chemical antifouling system by the marine macro-alga *Delisea pulchra* [154] was among the first identified QSIs. In 2002 and 2003, Hentzer et al. [12,13] performed subsequent studies of chemically modified halogenated furanones, thus delivering the first proof of concept regarding QS inhibition as an antimicrobial principle with the compound (**9**) C-30. The two mycotoxins, (**4**) Patulin and (**3**) penicillic acid, were later discovered as QSIs from *Penicillium* species [155] (See structures of the different compounds in Table 1). Patulin was showed to promote clearance of a *P. aeruginosa* infection in vivo as well as decreasing *P. aeruginosa* biofilm tolerance to tobramycin. Both C-30, patulin and penicillic acid are not applicable in a medical context because of toxic effects in combination with a possible carcinogenic property of C-30 [156,157,158]. These compounds, however, are to be considered experimental drugs that were used successfully to deliver proof of concept with regard to blocking QS as a viable antimicrobial strategy. Work on the development of chemically modified furanones is still ongoing. Non-brominated furanones, for example, have shown to be less cytotoxic [159].

These discoveries have led to an ongoing search for QSIs from both natural origin and synthetic designed compounds with the potential for medicinal application. However, the most widely used method towards especially *P. aeruginosa* QS has been incorporated through targeting the receptors by QSIs [160]. It is hypothesized that, by generating AHL analogs which fit the LuxR-homolog, a competitive and nonproductive signal-receptor complex is generated, in turn leading to disruption of further downstream signaling. By this method centered on AHL structural analogs, where either the lactone moiety or the acyl tail is varied, several studies have successfully identified a range of QS modulators. Studies of AHL analogs with non-native homoserine lactone rings and analogs with structural variations in the acyl chain have led to several very potent LasR antagonists and agonists [161,162,163,164,165]. By simply changing the length or degree of saturation of the alkyl chain, agonists with nanomolar activity have been discovered [165,166]. The crystal structure of the LasR protein have allowed for a computational and more rational design for the development or identification of new QS modulators [166,167], such as the identification of the three drugs (**12**) salicylic acid, (**11**) nifuroxazide, and chlorzoxazone through structure-based virtual screening [145]. An alternative approach is high-throughput screening, which gives the possibility of identifying compounds with a large structural variety. A few studies have documented a complete random screening of very large libraries. From an ultra-high-throughput screening of 200,000 small compounds, the two most active QSIs identified against LasR contained a 12-carbon alkyl tail, and thus resemble the 3-oxo-C12-HSL. The first compound designated (**6**) PD12 had an IC_50_ (the half maximum inhibitory concentration) of 30 nM, whereas the second, designated (**7**) V-06-018, had an IC_50_ of 10 μM. Both compounds inhibited the production of elastase and pyocyanin [168].

Several studies by others and us have identified different sulfur-containing compounds as QSIs. Garlic extract has previously been shown to have strong inhibitory effects on the *P. aeruginosa* QS system. In a nematode chronic infection model, garlic extract was shown to lower mortality to that of 5% of an untreated control, and in addition increased susceptibility of *P. aeruginosa* biofilms towards tobramycin treatments [169]. In a following study, Bjarnsholt et al. [170] showed a faster clearance of a *P. aeruginosa* infection in a pulmonary mouse model when treated with garlic extract. Garlic contains a number of active QSI compounds many of which were lost during the bioassay-guided fractionations and purification procedures. From a study with garlic extract, it was shown by thin layer chromatography **(**TLC) analysis to contain a minimum of three active QSI compounds [169], one of which had been previously identified. However, the compound only exhibited QSI activity in the *lux* system [171]. The major active QSI compound was identified to be (**1**) ajoene, a sulfur-rich small molecule produced from two allicin molecules which originate from aliin by an enzymatic process that occurs when garlic is crushed, chopped and/or heated. Ajoene exists as a mixture of two isomers, *E* and *Z* [172] and isolated and published for the first time by Block et al. [173]. Investigations of synthetic ajoene revealed a molecule capable of blocking the QS-regulated production of rhamnolipid. This rescues PMNs from being killed by *P. aeruginosa* biofilms as well as it lowered the tolerance of in vitro biofilm to tobramycin. In addition, the presence of eDNA in a *P. aeruginosa* biofilm was decreased with the increased concentration of ajoene added, which, in turn, corresponds with the lowered tolerance to tobramycin. In vivo studies of lung-infected mice showed significant reductions of the infections in the ajoene-treated mice.

From a screening of 69 different foods and plants, horseradish extract showed the highest QSI activity and by bioassay-guided fractionation, (**2**) iberin was identified as the active QSI, showing strong activity against *P. aeruginosa* [174]. Iberin, is an isothiocyanate containing a sulfinyl group such as ajoene. A QSI showing promising in vitro results is not a guarantee for a functional compound, in vivo. This became apparent with iberin treatment showing no significant decrease in infection using an intraperitoneal foreign-body infection mouse model. Reasons for the apparent loss of activity in vivo could be due to either the highly reactive isothiocyanate group, or the observed up-regulation (21- to 100-fold) of the *mexEF-oprN* operon encoding components of a resistance-nodulation division (NDR) efflux pump. The isothiocyanates cheirolin, iberverin, sulforaphane, and alyssin were also shown to have QSI activity against *P. aeruginosa*. However, they all possessed lower QSI activity compared with iberin. Another study also identified sulforaphane as a QSI, as well as the isothiocyanate erucin extracted from broccoli [175]. In addition, has (**8**) isothiocyanates been shown to covalently bind to the lasR pocket by computational conformational analysis [176]. Both horseradish and broccoli belongs to the family *Brassicaceae.* A recent study identified several sulfur-containing QSI compounds with activity in the nano molar range from a screening of a compound library combined with structure-activity relation studies to make analogs from positive hits [177].

The results from these studies suggest for a continuous search for potential sulfur-containing QSIs by both larger screenings of compound libraries and investigations of compounds with structural similarity to the different identified QSIs.

### 4.2. Compounds Targeting sRNAs

As mentioned previously, the strategies to identify new QSIs have been particularly focused on AHL structural analogs targeting the central parts of the QS system. However, recent studies have shown that the two sRNAs RsmY and RsmZ are possible targets leading to a modulating effect on QS regulated genes.

From transcriptomic analysis, it is apparent that the number of QS-regulated genes affected by the different QSIs varies a lot. The number of QS regulated genes down-regulated according to the QS regulon defined by Rasmussen et al. [155] by patulin (54), penicilic acid (147) [155], furanone C-30 (54) [12], and iberin (41) [174] are a significant high number compared to, e.g., ajoene that only down-regulates a minority, i.e., 11 genes [178]. The substantial difference in the number of genes affected may be due to different targets in the regulatory QS hierarchy. Ajoene has been identified as the major QSI present in garlic, which of course puts in question the difference in the number of genes targeted by ajoene and garlic extracts. An explanation to this disparity is likely the additional QSIs present in garlic extract, which may exhibit a variety of target specificities. An alternative explanation could be that other compounds are present in garlic, which protects ajoene from chemical modifications including degradation, thus improving its QS inhibitory efficacy.

From transcriptomic analyses of different QSIs such as C-30 [12], patulin, penicillic acid [155], iberin [174] and ajoene [178], none of the them show indications of targeting the central controllers of the QS system (*lasI*, *rhlI*, *lasR*, and *rhlR*). Investigations indicate that furanones target the QS system by causing the LuxR protein to degrade due to the formation of an unstable complex [179], whereas the specific target of patulin and penicillic acid has not been determined further. In a recent study we show that ajoene target the Gac/Rsm part of the QS system by lowering of the expression of the two small regulatory RNAs, RsmY and RsmZ [180] thereby removing the sequestering effect of the sRNAs on RsmA. In addition, the results show indications of a decrease in T6SS, suggesting that ajoene can change the mode of growth towards the acute infection state. Azithromycin and iberin has also recently been shown to function as a QSI by lowering the expression of *rsmY* and *rsmZ* [146,181]. As with ajoene, the specific mechanism to how this is obtained has not yet been elucidated. Comparing transcriptomic analysis of ajoene, iberin and azithromycin exposed bacteria, reveals a huge difference in the number of genes affected by the two compounds. Azithromycin in concentrations of 2 μg/mL has been found to down-regulate a total of 227 genes, 82 of which are QS-regulated [182], while iberin down-regulates 41 genes [174] according to the QS regulon defined by Rasmussen et al. [155]. In comparison, ajoene has been shown to only down-regulate 11 genes [178]. The difference in the number of genes targeted by the three QSIs is difficult to explain from the conviction that they share similar targets. However, apart from the differences between individual results these findings are all indications pointing towards *rsmY* and *rsmZ* as possible antimicrobial targets.

### 4.3. Compounds Modulating c-di-GMP Signaling

A large number of small molecule c-di-GMP inhibitors against different species have been published over the last decade, however, only c-di-GMP inhibitors of *P. aeruginosa* will be described in this review. The importance of DGCs in dispersion of biofilm together with the understanding that inhibition of PDEs would lead to biofilm formation has so far resulted in a focus to identify and develop inhibitors of DGCs. However, resent data suggesting that some PDEs only change local concentration of c-di-GMP with a following increase in virulence factors and no change in biofilm formation have extended the research to develop selective PDE inhibitors.

The first small compounds working as c-di-GMP inhibitors against *P. aeruginosa* were identified from a initial screening using a *Vibrio cholerae* c-di-GMP screen of 66,000 compounds and extracts of natural products [183]. From the initial screening several lead compounds (**13** and **14**) (Table 2) were found to reduce biofilm formation and identified as inhibitors of DGC activity in *P. aeruginosa*. The same group used an in silico pharmacophore-based screen of a focused library containing guanine-like compounds to identify four molecules, LP3134 (**15**), LP3145 (**16**), LP4010 (**17**) and LP1062, as potential DGC inhibitors. The compounds were shown to disperse *P. aeruginosa* biofilm through c-di-GMP inhibition and compound (**15**) were additionally found to inhibit *P. aeruginosa* adherence to a surface as well as to reduce biofilm on urethal catheters [184]. A recent study found ebselen (**18**) to inhibit c-di-GMP binding to DGS by covalently modulating cysteine residues on WspR in low μM concentrations and it was shown to affect c-di-GMP regulated phenotypes such as increasing swimming motility and decreasing biofilm formation [185]. Ebselen is a synthetic orgnoselenium compound that has previously been shown to modify cysteine residues as well as having multiple activities such as anti-inflammatory and anti-oxidant. In a recent study by us, the anti-cancerous drug doxorubicin (**19**) was shown to lower c-di-GMP levels in *P. aeruginosa* as well as decreasing expression of a number of genes related to biofilm formation. Doxorubicin was identified from a screening of 5000 compounds using the *cdrA* monitor recently developed by us. By quantification of the intracellular concentration of c-di-GMP using HPLCMS-MS, doxorubicin was shown to lower the c-di-GMP content approximately 50%. Surprisingly, doxorubicin was not able to change DGC activity of WspR and could not inhibit biofilm formation or dispersion per se, which was hypothesized to be a result of eDNA release from dead cells [186]. Not many studies have investigated the potential of using c-di-GMP analogs as inhibitors against *P. aeruginosa*. An investigation of different c-di-GMP analogs with 2′-modifications revealed that changing the hydroxyl group to a flourine group ((**20**) 2′-F-c-di-GMP) increased the binding affinity to the I-site of DGCs with four times compared to the native c-di-GMP [187]. A recent study showed the first example of a specific inhibitor of a *P. aeruginosa* c-di-GMP PDE to regulate virulence and not biofilm formation [188]. The benzoisothiazolinone compound (**21**) was identified from a high throughput docking of 250.000 compounds against the *E. coli* PDE YahA. The following in silico test revealed that the compound did not inhibit YahA, whereas it was shown to inhibit the PDE RocR from *P. aeruginosa* with no effect on biofilm formation.

## 5. Potential Treatment Strategies by Cellular Signaling Perturbation

Treatment of biofilm infections is significantly more difficult and complex compared to the relatively simple task of treating acute infections by general use of a single antibiotic. Even life-threatening, acute infections are usually treatable, if diagnosed and treated in time with the right combination of antibiotics. The complexity of treating biofilm-related infections is mainly due to previously described protective matrix components that offer increased tolerance towards most antibiotics, as well as to the PMNs of the innate immune system. Therefore, the prevention of biofilm formation is of great importance, and, incidentally, the human body often, and quite effectively, supports such prevention. In cases where biofilm formation is expected to develop with, e.g., CF patients, intermittent colonization is treated through early, aggressive eradication therapy to avoid biofilm formation. However, if a biofilm infection occurs, i.e., a chronic infection, the strategy changes to chronic suppressing antibiotic therapy; a combination treatment through high doses of antibiotics with different targets. This treatment goes on for an extended period. For a CF patient diagnosed with a chronic *P. aeruginosa*, this means for the rest of his or her life [3]. Surgical removal of the infected area is still the most efficient method with which to eradicate a biofilm infection. This method is used in cases were biofilms are present in e.g., catheters, implants, and/or wounds. For CF patients with both intermittent and chronic *P. aeruginosa* infections, inhalation of DNase, combined with antibiotic treatment, is used. This has been proven to reduce the viscosity, and perhaps also to destabilize the biofilm [3]. This can be explained by the removal of eDNA in the biofilm matrix.

As stated earlier, different strategies to overcome a biofilm infection have been studied intensively, not least the possibility of weakening the biofilm. In the following, the current status on using QSIs or c-di-GMP inhibitors for this purpose will be discussed. On the most general note, application of QSIs as treatment of *P. aeruginosa* biofilms has proven, from both in vitro and in vivo studies, to be a promising approach, and three statements from the investigations can be regarded as of special importance in the evaluation of the QSI strategy as a viable treatment method. First, a biofilm’s increased tolerance to aminoglycosides, such as tobramycin, is lowered. This is likely achieved due to a decrease in the production of eDNA as matrix component. Second, a biofilm’s increased tolerance towards PMNs is lowered. This is believed to be the result of a reduced production of rhamnolipid. Finally, by especially using mouse-models, treatment of *P. aeruginosa* infections with particular QSIs has proven to promote rapid clearing of the infection. This is likely explained by the decrease in the production of rhamnolipid, which, in turn, strengthens the host’s innate immune system in its antimicrobial activities. Moreover, it should be noted that combination treatment with tobramycin and different QSIs has showed a significant decrease in *P. aeruginosa* infection in an implant mouse model [189] and increased survival of infected *Caenorhabditis elegans* [190].

Several of the available antibiotics show biological activities against alternatively targets such as QS. Azithromycin does not inhibit growth of *P. aeruginosa* at the obtainable in vivo concentrations; however, it is still used routinely in the treatment of CF patients with a *P. aeruginosa* infection. This is because several trials have shown a general improvement in lung function [191]. The general understanding of this functionality of azithromycin is that it inhibits QS. Azithromycin, ceftazidime, and ciprofloxacin have been shown by Skindersoe et al. [182] to inhibit the expression of a range of QS-regulated virulence factors.

Potential future treatment of biofilm infections could include different strategies, such as, single, combination and prophylactic (single or combination treatment) with QSI or c-di-GMP inhibitor drugs. Single treatment with QSIs would probably only be beneficial depending on the patient’s antimicrobial efficacy of the immune system and would therefore not be functional in immunocompromised patients. The same issues are relevant regarding c-di-GMP inhibitors. Larger scale dispersal of bacteria from a biofilm infection following treatment with a c-di-GMP inhibitor may induce conditions of sepsis and potentially become lethal to the patient.

Consequently, it is primarily combination-treatment strategies which seem likely to show a beneficial outcome in the treatment of biofilm related infections. Prophylactic treatments may keep the infection in an acute state which should be expected to be more susceptible to treatments with our present assortment of antibiotics. However, presently, no studies with investigations into combination of different QSIs and c-di-GMP inhibitors have been published.

## 6. Final Remarks

Some 50 years ago, in the golden age of antibiotic discovery, bacterial infections were predominantly acute (as they still are in underdeveloped countries). However, major societal changes such as rises in the elderly population and hospitalization rates increasingly expose citizens to chronic infections. A major shortcoming of most antibiotic discovery is that it aims to achieve growth inhibition of bacteria present in an unshielded state. However, biofilms predominantly consist of shielded cells in a non-growing state. Furthermore, when new resistance mechanisms emerge, they spread readily by cell-to-cell contact in the densely packed biofilms. Since planktonic bacteria are easier to kill, as they grow actively and un-shielded, the aim of novel treatment scenarios would be to dismantle the biofilm, liberate the bacteria and then kill them with antibiotics and the efficacy of the innate immune system. Many molecular tools have been developed to identify and investigate small molecules for inhibitory activity which has resulted in the identification of a large number of potential compounds. Of the identified active compounds, many show excellent in vitro activity, and, with this acknowledgement, the next step in this scientific area is to move closer to investigate the potentials of using this therapeutic approach in a more clinical setting. Several scientific issues are important to incorporate in the investigations to further develop the potentials of using this approach as a future treatment method of biofilm infections. Firstly, natural sources have proven a good place to search for inhibitors, and in which to find lead structures. This, of course, is based on the presence of a very diverse and rich selection of secondary metabolites. However, many studies of extracts from natural sources with inhibitory activity do not identify the molecular structures of the active molecules. This, however, is important to gain knowledge of the molecule’ to identify new potential lead structures’ and to investigate the specific activity and potentially unwanted side effects of the active compound. Secondly, in vivo experiments are crucial in the search for potential drug candidates because many unforeseen reasons can diminish the effect of a molecule in an animal study, thus removing the relevance for testing it in a clinical trial. Unfortunately, many studies of inhibitors do not employ in vivo investigations.

## Figures and Tables

**Table 1:** Examples of inhibitors of the *N*-acyl-l-homoserine lactone (AHL) and (*Pseudomonas* quinolone signal) PQS part of the quorum sensing system and/or the Gac/Rsm cascade by *rsmY* and *rsmZ* inhibition from natural or synthetic sources as well as commercial drugs against *P. aeruginosa.*

Compounds	Structure	AHL ^a^	Pqs ^a^	rsmY/rsmZ ^b^	In Vivo ^c^	References
**Natural Sources**						
(**1**) Ajoene	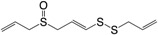	+	+ ^d^	+	+	[178,180]
(**2**) Iberin	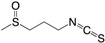	+	+	+	÷	[146,174]
(**3**) Penicillic acid	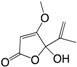	+	NI	÷ ^d^	NI	[155]
(**4**) Patulin	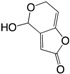	+	NI	÷ ^d^	+	[155]
Synthetic sources						
(**6**) PD12	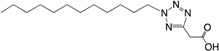	+	NI	NI	NI	[168]
(**7**) V-06-018	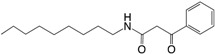	+	NI	NI	NI	[168]
(**8**) Isothiocyanates	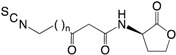	+	NI	NI	NI	[176]
(**9**) C-30	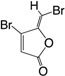	+	÷	÷ ^d^	+	[12]
Commercial drugs						
(**10**) Azithromycin	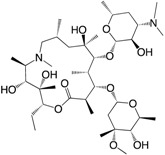	+	NI	+	+	[181,182]
(**11**) Nifuroxazide	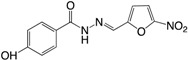	+	+	NI	NI	[145]
(**12**) Salicylic acid	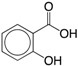	+	+	NI	NI	[145]

^a^ Inhibition of the AHL and/or the PQS system measured by reporter systems; ^b^ Inhibition of the sRNAs, rsmY and rsmZ measured by reporter systems; ^c^ Compounds tested in a in vivo model with (+) or with out (÷) an effect to lower infection level; ^d^ Unpublished data. NI: Not investigated; +, investigated with positive effect; ÷, investigated with no effect.

**Table 2:** Representative non-nucleotide and nucleotide small molecule inhibitors of *Pseudomonas aeruginosa* diguanylate cyclase (DGC) and phosphodiesterase (PDE).

Compound	Structure	WspR IC_50_ (μM)	References
DGC inhibitors			
Non-nucleotide			
(**13**) DI-4	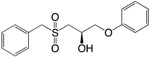	8.17	[183]
(**14**) DI-10	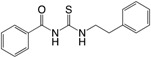	12.2	[183]
(**15**) LP3134	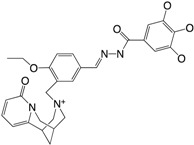	44.9	[184]
(**16**) LP3145	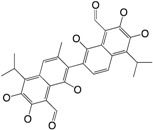	70.93	[184]
(**17**) LP4010	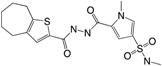	102.4	[184]
(**18**) Ebselen	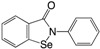	13.6	[185]
(**19**) Doxorubicin	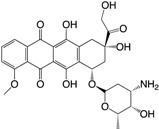	÷ ^a^	[186]
Nucleotide			
(**20**) 2′-F-c-di-GMP	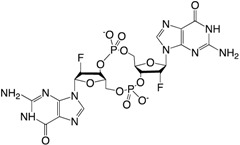	NI	[187]
PDE inhibitors			
Non-nucleotide			
(**21**) Compound	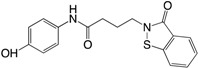	NI	[188]

^a^ Decrease in expression of *cdrA.* NI: Not investigated; ÷, Investigated with no effect.
